# *In silico* functional and pathway analysis of risk genes and SNPs for type 2 diabetes in Asian population

**DOI:** 10.1371/journal.pone.0268826

**Published:** 2022-08-29

**Authors:** Md. Numan Islam, Md. Golam Rabby, Md. Munnaf Hossen, Md. Mostafa Kamal, Md. Ashrafuzzaman Zahid, Md. Syduzzaman, Md. Mahmudul Hasan

**Affiliations:** 1 Department of Nutrition and Food Technology, Jashore University of Science and Technology, Jashore, Bangladesh; 2 Department of Immunology, Health Science Center, Shenzhen University, Shenzhen, China; 3 Division of Plant Science, University of Missouri, Columbia, Missouri, United States of America; Centre de Recherche des Cordeliers, FRANCE

## Abstract

Type 2 diabetes (T2D) has earned widespread recognition as a primary cause of death, disability, and increasing healthcare costs. There is compelling evidence that hereditary factors contribute to the development of T2D. Clinical trials in T2D have mostly focused on genes and single nucleotide polymorphisms (SNPs) in protein-coding areas. Recently, it was revealed that SNPs located in noncoding areas also play a significant impact on disease vulnerability. It is required for cell type-specific gene expression. However, the precise mechanism by which T2D risk genes and SNPs work remains unknown. We integrated risk genes and SNPs from genome-wide association studies (GWASs) and performed comprehensive bioinformatics analyses to further investigate the functional significance of these genes and SNPs. We identified four intriguing transcription factors (TFs) associated with T2D. The analysis revealed that the SNPs are engaged in chromatin interaction regulation and/or may have an effect on TF binding affinity. The Gene Ontology (GO) study revealed high enrichment in a number of well-characterized signaling pathways and regulatory processes, including the STAT3 and JAK signaling pathways, which are both involved in T2D metabolism. Additionally, a detailed KEGG pathway analysis identified two major T2D genes and their prospective therapeutic targets. Our findings underscored the potential functional significance of T2D risk genes and SNPs, which may provide unique insights into the disease’s pathophysiology.

## Introduction

Type 2 diabetes mellitus (T2DM) is a devastating global disease and one of the most prevalent metabolic disorders [1, 2]. T2DM has a convoluted etiology that involves both unchangeable risk factors such as age, genetics, race, and ethnicity and modifiable risk factors including physical activity, smoking, and diet. Diabetic patients are fast rising in number as a result of an increase in the incidence and prevalence of obesity, which is mostly attributed to western cuisine and other lifestyle choices [3]. Patient numbers have quadrupled in the last three decades, making it a significant global public health concern [4]. Increases in the incidence of risk factors such obesity, hypertension, cardiovascular disease and prediabetes, as well as an aging population, are assumed to be contributing to the rising prevalence of diabetes [5, 6]. Increased screening, as well as changes in diagnostic criteria, may lead to increased diabetes prevalence, along with changes in risk factor prevalence. Worldwide, 425 million persons (1 in 11) have diabetes, 90% of which is type 2. (T2DM) [7, 8].

T2D has gained notoriety as a leading cause of death, disability, and rising healthcare expenses. There is strong evidence that genetic factors have a role in the development of T2D [9]. Several strong T2D single nucleotide polymorphisms (SNPs) were found in recent waves of genome-wide association studies (GWASs) across the European and Asian regions, even though these SNPs explained only a small percentage of T2D heritability in aggregate [10–12]. Because of their effect on the expression of their target genes, SNPs in the protein-coding region have been related to hundreds of diseases. Furthermore, noncoding regions account for more than 90% of variants discovered in GWAS [13], and the mechanisms through which these SNPs contribute to illness risk are mysterious. The role of SNP-related genes connected to type 2 diabetes in Asian people, is still unknown. Additionally, genes and transcription factors are a critical component in the pathophysiology of T2D. According to research, transcription factors may influence pancreatic cell growth, β-cell function, and T2D-related signaling pathways [14, 15]. Previous studies have demonstrated that various genes associated with type 2 diabetes are found in the leptin signaling pathways, including signal transducer and activator of transcription 3 (STAT3), which acts as a regulator of the signal transduction of various cytokines, growth factors, and hormones involved in the regulation of body growth and immune responses. Additionally, STAT3 is a well-known regulator of insulin resistance [16, 17]. While research into the pathophysiology of diabetes is progressing, we still need to understand the critical genetic features that directly regulate T2D.

The purpose of this study was to select the genes and SNPs linked with T2D risk in the Asian region using GWAS data and to estimate their molecular and biological function in T2D pathogenesis. We employed bioinformatics analysis to further explain the potential functional importance of T2D-associated SNPs in the etiology of T2D by combining T2D-associated SNPs and their target genes from GWAS data for the Asian region. Additionally, we also investigated the possible risk gene pathway and genes-drugs interaction. Our findings may shed new light on the genetic origins of type 2 diabetes and their possible function in T2D pathogenesis.

## Materials and methods

### T2D GWAS datasets and associated gene and SNPs selection

We retrieved potential genes and SNPs associated with type 2 diabetes in Asian ancestry from publicly available GWAS datasets (https://www.ebi.ac.uk/gwas/) and GWAS studies done by Spracklen *et al*., [12] and Kooner *et al*., [10] and these two studies represent the summery of GWAS study in Asian region. We have selected potential SNPs using the GWAS significance threshold (p-value < 5×E-5). Following that, we used proxy SNAP (https://www.broadinstitute.org/mpg/snap/ldsearch.php) to identify SNPs in linkage disequilibrium (LD) that were associated with T2D SNPs retrieved for east and south Asian previously. The inclusion criteria for LD SNPs were as follows: a distance of 500 KB from the query SNP with a pairwise LD coefficient of >0.8 [10, 12]. Finally, a total of 22 genes and 23 potential SNPs were selected and further used for their functional prediction and possible underlying molecular mechanism related to T2D in Asian population.

### Functional prediction of T2D associated SNPs

To investigate the functional consequences of T2D associated SNPs, we used HaploReg v4.1, a comprehensive resource that incorporates data from Roadmap Epigenomics, ENCODE, and the Genotype-Tissue Expression Project (GTEx), among others, to examine the effect of T2D associated SNPs on gene expression (eQTLs) and regulatory motif alterations within sets of genetically linked T2D risk SNPs. Additionally, we conducted a transcription factor enrichment study using the SNP2TFBS (http://ccg.vital-it.ch/snp2tfbs/) program, which allows users to choose and display variations affecting single or many transcription factors [18].

### Gene ontology (GO) analysis

To get a better understanding of the targeted gene’s biological significance and to decipher biological networks, we ran a gene ontology (GO) enrichment study. ToppGene (https://toppgene.cchmc.org/) was used to presume key molecular activities and biological processes that may provide light on the pathogenesis of the disease under investigation. For this study, the ToppGene Suite portal’s default settings were used, which means that the FDR was adjusted and P<0.05 was considered statistically significant [19, 20].

### Protein-protein interaction networks

STRING v11.0 (https://string-db.org/) is a database of recognized and anticipated protein interactions, including associations of physical and functional properties. It combines interaction data statistically obtained from the genetic context, high-throughput studies, co-expression, and prior information. To investigate the functional association and interaction of T2D-associated SNPs, we created gene (protein) interaction networks using the STRING v11.0 database’s default settings (observed interaction, 70; expected interaction, 8.83; Benjamini adjust p-value < 1×E-10; proteins,76) [21, 22]. We used the Markov Clustering Algorithm (MCL) to identify clusters of proteins in the STRING augmented and extended networks. The algorithm was executed using an inflation parameter of 2 to obtain a reasonable level of granularity in the protein clusters [23].

### KEGG pathway analysis

The Kyoto Encyclopedia of Genes and Genomes (KEGG) is a reference knowledge used to analyze genomic sequences and other high-throughput data for biological purposes [24]. At the molecular and higher levels, KEGG provides functional meaning to genes and genomes. We used The Database for Annotation, Visualization, and Integrated Discovery (DAVID) to obtain the pathways and which allows for a comprehensive analysis of high-throughput gene functions [25].

### Key gene-drug interaction network analysis

The Comparative Toxicogenomic Database was used to design the key gene-drug interaction network for chemotherapeutic drugs that maybe reduce or elevate the mRNA or protein expression levels of the key genes [26]. The CTD attempts to develop an understanding of the interactions between environmental chemicals and genes, along with their impact on human health. Basically, the CTD systematic search was conducted for PAX4 and HNF4A key genes, and the gene-drug interaction networks were visualized using Cytoscape version 3.8.2 [27].

## Results

### Functional prediction of T2D associated SNPs

We began our investigation by identifying 22 candidate genes and 23 related SNPs from a GWAS study conducted in the Asian area (S1 Table in S1 File) and identified candidate SNPs based on their GWAS significance (p-value <5×E-5). To ascertain the possible functional consequences of selected T2D risk SNPs, we examined their influence on regulatory motifs and gene expression. Following annotation, it was discovered that 23 T2D risk SNPs altered the transcription factor binding motif, highlighting the significant regulatory potential of T2D risk SNPs (Table 1). Additionally, the analysis highlighted 16 T2D risk SNPs that were associated with eQTL evidence.

**Table 1:** Functional prediction of type 2 diabetes associated SNPs in Asian populations.

Chr	Pos (hg38)	Variant	Motifs Changed	Selected eQTL Hits	GENCODE Genes	dbSNP Functional Annotation
15	62104190	rs7172432	7 altered motifs	1	33kb 3’ of C2CD4A	-
3	64062621	rs831571	PLZF	-	5.3kb 5’ of RP11-129B22.1	-
4	1316113	rs6815464	6 altered motifs	-	MAEA	intronic
6	38139068	rs9470794	Ascl2,BHLHE40,Myf	1	ZFAND3	intronic
6	39316274	rs1535500	5 altered motifs	9	KCNK16	missense
7	127524904	rs6467136	5 altered motifs	1	39kb 3’ of AC000124.1	-
9	4287466	rs7041847	Osr	-	GLIS3	intronic
19	33402102	rs3786897	LF-A1,SP1,SZF1-1	4	PEPD	intronic
20	44318326	rs6017317	CEBPA,Mef2,STAT	4	7.2kb 5’ of FITM2	-
8	41661944	rs515071	4 altered motifs	3	RP11-930P14.1	intronic
10	119389891	rs10886471	-	-	GRK5	intronic
15	38530704	rs7403531	Hmbox1	22	RP11-275I4.1	intronic
7	127606849	rs10229583	KAP1,Maf,TCF12	1	3.4kb 3’ of PAX4	-
9	136357696	rs11787792	Pax-5,TCF12,ZNF263	13	GPSM1	intronic
17	7037074	rs312457	4 altered motifs	-	SLC16A13	intronic
3	186948673	rs16861329	RXRA,TBX5	-	ST6GAL1	intronic
10	69171718	rs1802295	5 altered motifs	9	VPS26A	3’-UTR
15	77454848	rs7178572	THAP1	4	HMG20A	intronic
15	89831025	rs2028299	Egr-1,Ets,Znf143	30	AP3S2	3’-UTR
20	44360627	rs4812829	Mef2,Nkx2	2	HNF4A	intronic
13	23290518	rs9552911	6 altered motifs	-	SGCG	intronic
2	134722410	rs6723108	Foxp3,Pou5f1,STAT	10	3.4kb 5’ of TMEM163	-
2	164645339	rs3923113	HEN1	1	8.3kb 3’ of COBLL1	-

**Fig 1:**
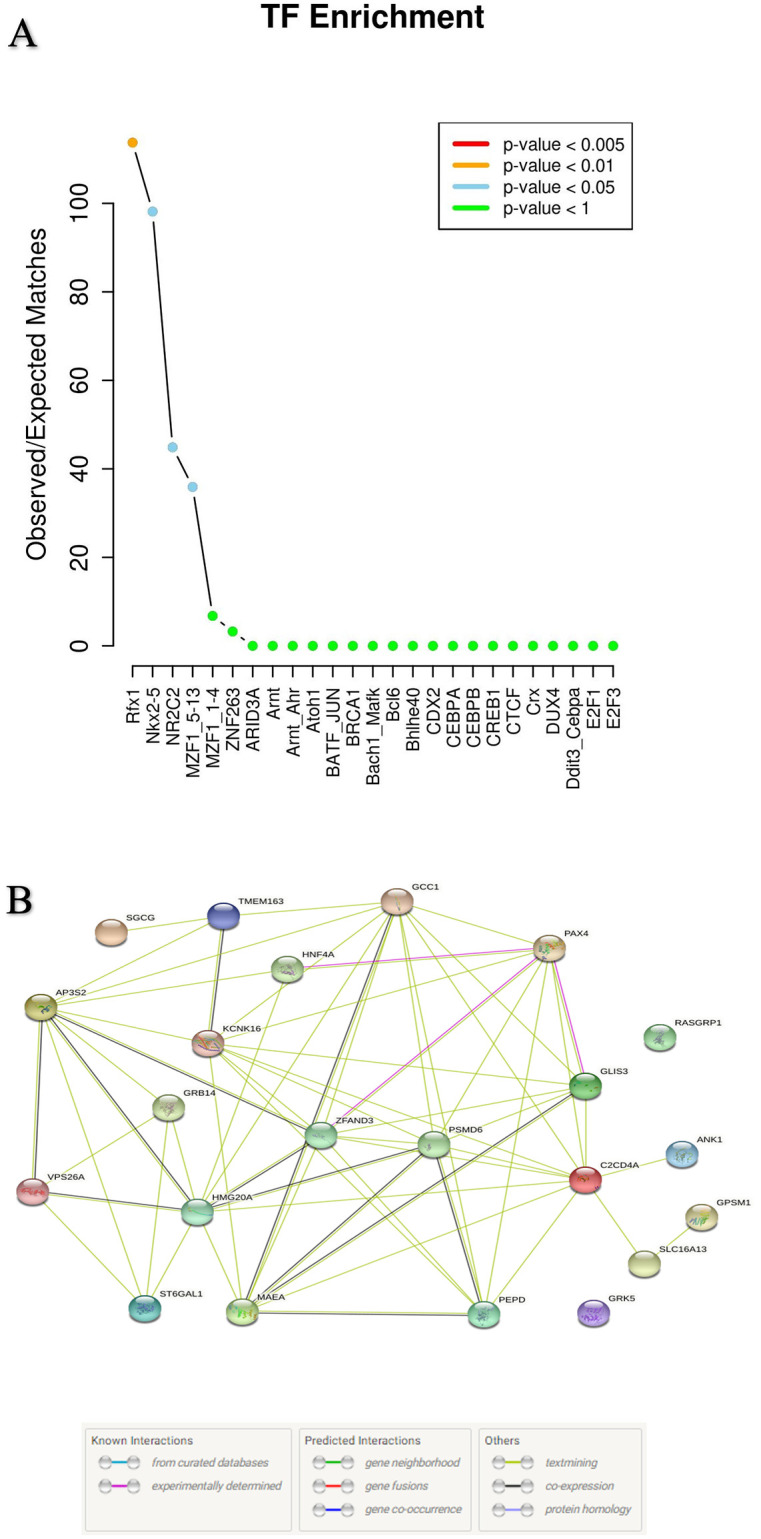
Functional prediction and protein-protein interaction of T2D risk genes and SNPs. (A) Transcription factors (TF) enrichment, TFs are sorted based on their enrichment. (B) Protein-protein interaction of T2D risk genes. In protein-protein interaction, connections are based on co-expression and experimental evidence. Each filled node denotes a gene; edges between nodes indicate protein-protein interactions between protein products of the corresponding genes. Different edge colors represent the types of evidence for the association.

However, the effect of T2D risk SNPs on the transcription binding affinity was determined and analysis results reported top four enrichment transcription factors and these are Rfx1, Nkx2-5, NR2C2 and MZF1_5–13 (Fig 1A). Fig 1A shows the 25 transcription factors that interact with identified SNPs. According to p-values, these SNPs were substantially enriched for disruption of four TFs, including Rfx1 (P = 0.008), Nkx2-5 (P = 0.01), NR2C2 (P = 0.022), and MZF1_5–13 (P = 0.027) (S2 Table in S1 File). The analysis showed that these four transcription factors (TF) are highly significant for T2D risk genes and SNPs. Instead, other TFs are poorly associated with T2D risk genes and SNPs.

### GO analysis of T2D associated SNPs target genes

In order to thoroughly explore if the specific T2D-related SNPs are specifically correlated with T2D, we performed a GO analysis. Interestingly, we observed a significant molecular functions and biological processes (Table 2). The GO term beta-adrenergic receptor kinase activity, zinc ion binding, beta-adrenergic receptor kinase activity, transition metal ion binding, and Long-chain fatty acyl-CoA binding may have a crucial role in T2D pathogenesis. In this study, GO analysis reported that SNPs are may responsible for binding more stearic acid in serum and impaired beta cell function.

**Table 2:** The most significant gene ontology (GO) terms for type 2 diabetes associated SNPs target genes.

	ID	Name	p-value	Genes from Input	Genes in Annotation
Molecular Functions	GO:0102009	Proline dipeptidase activity	9.80E-04	1	1
	GO:0102010	Beta-galactoside alpha-2,6-sialyltransferase activity	1.96E-03	1	2
	GO:0102011	Stearic acid binding	1.96E-03	1	2
	GO:0102012	Transition metal ion binding	3.88E-03	5	1130
	GO:0102013	Beta-adrenergic receptor kinase activity	3.91E-03	1	4
	GO:0102014	G protein-coupled receptor kinase activity	6.83E-03	1	7
	GO:0102015	Long-chain fatty acyl-CoA binding	7.80E-03	1	8
	GO:0102016	Arachidonic acid binding	7.80E-03	1	8
	GO:0102017	Zinc ion binding	8.32E-03	4	849
	GO:0102018	Icosatetraenoic acid binding	8.78E-03	1	9
Biological Processes	GO:1904145	Negative regulation of meiotic cell cycle process involved in oocyte maturation	9.90E-04	1	1
	GO:0031018	Endocrine pancreas development	1.75E-03	2	63
	GO:1902569	Negative regulation of activation of Janus kinase activity	1.98E-03	1	2
	GO:0062111	Zinc ion import into organelle	1.98E-03	1	2
	GO:0099180	Zinc ion import into synaptic vesicle	1.98E-03	1	2
	GO:1903537	Meiotic cell cycle process involved in oocyte maturation	2.97E-03	1	3
	GO:1903538	Regulation of meiotic cell cycle process involved in oocyte maturation	2.97E-03	1	3
	GO:0002528	Regulation of vascular permeability involved in acute inflammatory response	3.95E-03	1	4
	GO:0031016	Pancreas development	4.26E-03	2	99
	GO:0006520	Cellular amino acid metabolic process	4.53E-03	3	349

In biological function, GO analysis shows that selected SNPs may negatively regulate the activation of Janus kinase activity (JAK) and meiotic cell cycle process involved in oocyte maturation (Table 2). Study results also stated that T2D-associated SNPs may regulate endocrine pancreas development and more studies are needed to describe the underlying function of risk SNPs to pancreas development. Insulin signaling (IS) is very crucial for glucose homeostasis and its directly regulated oocyte growth and maturation [28]. On the other hand, GO analysis reported that the risk SNPs are negatively regulated of meiotic cell cycle process involved in oocyte maturation. Other interesting GO terms, for instance proline dipeptidase activity, long chain fatty acyl-CoA binding, and cellular amino acid metabolic process, may have a potential function in the T2D pathogenesis.

### Protein-protein interaction networks (PPI)

To partially describe the functional relationships and interaction networks among the 22 T2D-linked SNPs target genes, we analyzed the protein-protein interaction patterns of T2D risk genes. Fig 1B indicated a substantial correlation between the topological characteristics and biological function of T2D associated SNPs target genes. ZFAND3, PAX4, HNF4A, PSMD6, MAEA, KCNK16, GCC1, GLIS3, VPS26A, HMG20A, and AP3S2 are the genes with the strongest interactions. Importantly, our results described that interactions among the PAXA, HNF4A, ZFAND3 and GLIS3 proteins are experimentally determined. Furthermore, this network of the 22 T2D-associated proteins had more interactions among themselves than would be expected for a set of proteins of similar size, randomly selected from the human genome. Such an enrichment indicates that the proteins are functionally connected as a group. Interestingly, these proteins are implicated in the energy reserve metabolic process, glucose homeostasis, and negative regulation of pancreatic cell apoptosis, indicating that they may have a role in the etiology of T2D [29, 30].

### KEGG pathway analysis

**Fig 2:**
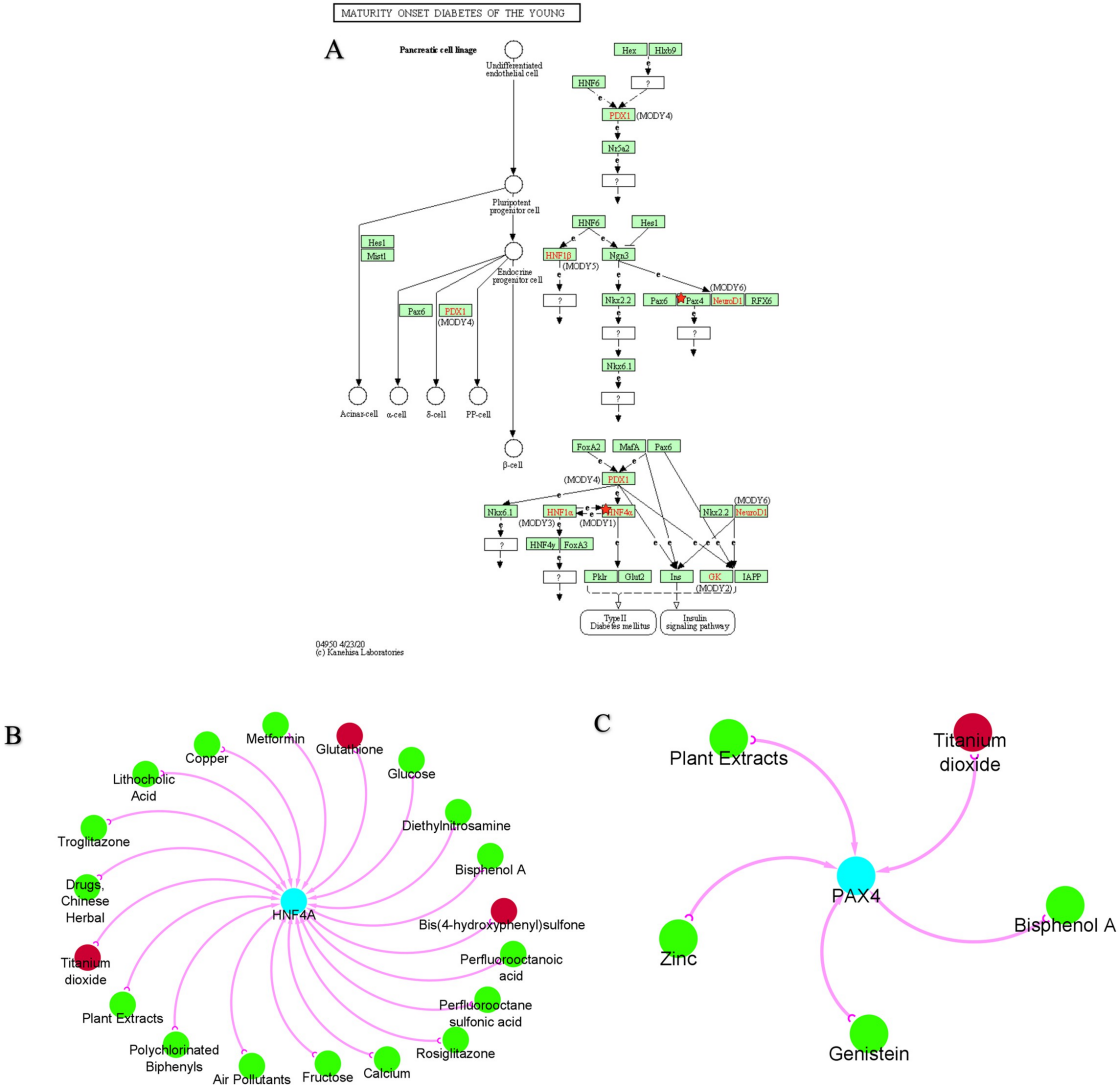
Key genes pathway identification and potential drug design. (A) KEGG pathway enrichment. PAX4 and HNF4A genes were significantly enriched in the maturity onset diabetes of the young. (B and C) HNF4A and PAX4 are each linked to different potential drugs. **Green node**: the drug decreases the key gene expression; and **red node**: the drug increases the expression of the key gene.

To elucidate the important genes, the KEGG pathways of these 22 genes were investigated. Several genes were found to be enriched, however hepatocyte nuclear factor 4 alpha (HNF4A) and paired box 4 (PAX4) were found to be strongly related to the diabetes onset pathway (Fig 2A). Additionally, the results reveal the presence of another subset of HNF1A, however this gene is not as significantly vulnerable as HNF4A. Additionally, HNF4A is associated with two molecular factors (Pklr and Glut2) involved in the pathophysiology of T2D. In comparison, the critical molecular component for PAX4 remains unknown.

### Interaction analysis of the key genes and drugs

The key gene-drug interaction network was established using to investigate the interaction between key genes and their interaction with chemical compounds for therapeutic targets of T2D. A variety of drugs, as depicted in Fig 2B and 2C, may affect the expression of these two key genes, PAX4 and HNF4A. These major transcription factor-drug interaction networks not only reveal which chemicals inhibit these key transcription factors and essential genes, but also how these genes may increase or reduce chemotherapeutic drug susceptibility.

Titanium dioxide, glutathione, and bis (4-hydroxyphenyl) sulfone all have the potential to promote HNF4A expression, whilst the remaining medicines have the potential to reduce HNF4A expression (Fig 2B). All of the medications or compounds in Fig 2C decrease PAX4 expression, with the exception of titanium dioxide, which increases it.

## Discussion

There are far more genetic variants in noncoding regions of the human genome than in protein-coding regions. There are several critical functional regulatory elements, such as SNPs, that may be critical in disease metabolism. In this study, we used publicly available GWAS datasets to identify 22 candidate genes and 23 single nucleotide polymorphisms (SNPs) associated with type 2 diabetes in Asians. Additionally, we examined the functional consequences of these SNPs and their target genes, conducted transcription factor enrichment analysis, GO and protein-protein interaction network studies, and examined pathways and significant gene-drug interaction networks.

Our findings demonstrated the probable function of 22 genes and 23 SNPs associated with type 2 diabetes in Asians. Intriguingly, functional prediction analysis revealed that 22 SNPs associated with type 2 diabetes altered the transcription factor binding motif, indicating the enormous regulatory potential of T2D risk SNPs (Table 1). Additionally, the research identified sixteen SNPs related to T2D risk that were connected with eQTL evidence. Additionally, the results suggested that the potential genes based on eQTL hits. KCNK16, HNF4A, AP3S2, VPS26A, TMEM163, GPSM1, RP11, and HMG20A are all potential genes. Recent research indicates that mutations in the HNF4A gene are involved in progressive β-cell dysfunction and hyperinsulinism, which can result in the establishment of early diabetes [31]. Additionally, the study discovered that the KCNK16 mutation is connected with beta cell activity and enhances TALK-1’s functional potential. TALK-1 inhibits glucose-induced depolarization of the membrane potential and Ca^2+^ influx [32]. AP3S2 and GPSM1 have also associated to diabetes and other metabolic disorder by impairing the function of Golgi vesicles formation and trafficking to lysosomes and cell signaling respectively [15]. Another interesting HMG20A gene was also reported as a risk gene for T2D. The mutation of this gene may regulate transcription factor; histone methylation [15].

Likewise, to investigate the effect of T2D risk SNPs on transcription binding affinity revealed that the top four transcription factors associated with enrichment are Rfx1, Nkx2-5, NR2C2, and MZF1 5–13. TF Rfx1is playing downstream role on signal transducer and activator of transcription 3 (STAT3) pathway while the expression of Rfx1 is regulated by interleukin-6 (IL-6)-STAT3 signaling pathway [33], and STAT3 plays an important role in diabetes pathogenesis [34]. TF Nkx2-5 regulates WNT signaling pathway [35] and interestingly, WNT signaling is associated with insulin activation [36]. On the other hand, NR2C2 is linked with insulin resistance [37].

The GO term beta-adrenergic receptor kinase activity has a crucial role in increasing insulin resistance which may promote diabetes pathogenesis [38]. High stearic acid in serum is responsible for inducing more lipotoxicity and decreasing insulin production from beta cells [39]. The GO analysis of T2D risk SNPs target genes confirmed the well-characterized JAK signaling pathways, but also identified numerous additional mechanisms likely involved in T2D pathogenesis, such as positive regulation of lipid metabolic processes and negative regulation of inflammatory response. It is well established that JAK signaling is critical for cytokine generation and immune homeostasis regulation [40]. Additionally, JAK signaling dysregulation may play a role in the development of obesity and diabetes [41].

A network analysis of protein-protein interactions discovered numerous hub genes that interacted and were responsible for T2D associations [42]. Then, we analyzed the possible pathway of T2D-associated genes and found that two key genes (PAX4 and HNF4A) are significantly responsible for T2D pathogenesis. PAX4 inhibited the expression of insulin and glucagon promoters and decreased the amounts of transcripts encoding genes necessary for β-cell function, proliferation, and survival. The viability of β-cells overexpressing either PAX4 R192H or PAX4 P321H, or both, was reduced under glucotoxicity stress conditions [43]. Thus, these PAX4 gene mutation may raise the risk of T2D by impairing target gene transcription control and/or reducing β-cell survival in high glucose conditions. Mutations in the genes encoding hepatocyte nuclear factor (HNF) 1A and 4A result in a monogenic form of diabetes called maturity-onset diabetes of the young (MODY). MODY is primarily caused by a defect in pancreatic β-cells’ glucose-stimulated insulin production, highlighting the critical functions of HNF1A and HNF4A in -cells [44]. Numerous large-scale genetic investigations have established those common variations of the HNF1A and HNF4A genes are also associated with type 2 diabetes, implying that they play a role in the etiology of both disorders [45, 46]. Recent experimental research has demonstrated that HNF1A regulates both the activity and proliferation of β-cells via the regulation of target genes such as glucose transporter 2, pyruvate kinase, collectrin, hepatocyte growth factor activator, and HNF4A. In comparison, HNF4A is primarily involved in regulating the function of β-cells [47].

Furthermore, to gain a better understanding of gene-targeted therapy, we investigated the interactions of essential genes with commonly used therapeutic agents. The results indicated that certain medications had the ability to modulate the expression of two critical genes. As shown in result section, titanium dioxide, glutathione, and bis (4-hydroxyphenyl) sulfone all have the ability to increase HNF4A expression, whilst the remaining medications have the potential to decrease it (Fig 2B). Except for titanium dioxide, which promotes PAX4 expression, all of the drugs or chemicals in Fig 2C inhibit PAX4 expression. Although additional research is need to unravel the precise gene-drug interactions.

In the current study, we conducted in-depth bioinformatics analysis to establish the functional significance of the T2D risk SNPs and genes in Asian region. However, our study highlighted four transcriptional factors, essential signaling pathways and some targeted genes and possible targeted drugs. Nevertheless, the databases involved in these bioinformatics investigations are limited; for example, we focus exclusively on genes and SNPs with Minor allele frequency (MAF > 0.001) from the GWAS study. We acknowledge that the functional prediction analysis overlooked a number of known variations fulfilling the MAF requirement. Furthermore, because functional annotation results are entirely dependent on computationally anticipated regulatory characteristics, model and algorithm selection is crucial for this type of research. As a result of the potential overshadowing of the functional significance of these candidate T2D risk genes and SNPs, additional experiment validation should be conducted to establish the functional mechanism of these prospective T2D risk genes and SNPs.

In summary, we identified four major transcription factors associated with diabetes pathogenesis in the Asian population by predicting the function of T2D risk genes and SNPs. Additionally, comprehensive bioinformatics study demonstrated that T2D risk genes and SNPs have significant molecular and biological functions which regulate diabetes pathogenesis. Furthermore, analysis revealed the two critical genes pathway and their prospective target medications. While exact mechanisms have to be investigated in vivo and in vitro, our findings provide new insights into how to improve the treatment and prognosis of T2D patients targeting genetics factors.

## Supporting information

S1 File Single nucleotide polymorphisms (SNPs) associated with type 2 diabetes in Asian populations and transcription factor enrichment (See details in the supporting information as S1 and S2 Tables, respectively). (DOCX)
