# Supplementary material for: In silico functional and pathway analysis of risk genes and SNPs for type 2 diabetes in Asian population
Source: PLoS One. 2022 Aug 29;17(8):e0268826. doi: 10.1371/journal.pone.0268826 (PMC9423640; doi:10.1371/journal.pone.0268826)
Supplement: S1 File — (DOCX) [file pone.0268826.s001.docx]

***In silico* functional and pathway analysis of risk gene and SNPs for type 2 diabetes in Asian population**

Md. Numan Islam^1^, Md. Golam Rabby^1^, Md. Munnaf Hossen^1,^ ^2^, Md. Mostafa Kamal^1^, Md. Mahmudul Hasan^1, 3^*

^1^Department of Nutrition and Food Technology, Jashore University of Science and Technology, Jashore-7408, Bangladesh

^2^Department of Immunology, Health Science Center, Shenzhen University, Shenzhen, China

^3^Division of Plant Science, University of Missouri, Columbia, MO, USA.

***Corresponding author’s Email address**: [hasanm_agb@yahoo.com](mailto:hasanm_agb@yahoo.com)

Table S1. Single nucleotide polymorphisms (SNPs) associated with type 2 diabetes in Asian populations

| **Sl No** | **Locus/**  **Gene** | **Ensemble ID** | **SNP** | **Chr** | **Allele (+/−)** | **RAF (Risk Allele Frequency)** | **OR (Odds Ratio)**  **(95% CI)** |
| --- | --- | --- | --- | --- | --- | --- | --- |
| 1 | *C2CD4A* | ENSG00000198535 | rs7172432 | 15 | A/G | 0.59 | 1.12 (1.08–1.16) |
| 2 | *PSMD6* | ENSG00000163636 | rs831571 | 3 | C/T | 0.61 | 1.09 (1.06–1.12 |
| 3 | *MAEA* | ENSG00000090316 | rs6815464 | 4 | C/G | 0.58 | 1.13 (1.10–1.16) |
| 4 | *ZFAND3* | ENSG00000156639 | rs9470794 | 6 | C/T | 0.27 | 1.12 (1.08–1.16) |
| 5 | *KCNK16* | ENSG00000095981 | rs1535500 | 6 | T/G | 0.42 | 1.08 (1.05–1.11) |
| 6 | *GCC1* | ENSG00000179562 | rs6467136 | 7 | G/A | 0.79 | 1.11 (1.07–1.14) |
| 7 | *GLIS3* | ENSG00000107249 | rs7041847 | 9 | A/G | 0.41 | 1.10 (1.07–1.13) |
| 8 | *PEPD* | ENSG00000124299 | rs3786897 | 19 | A/G | 0.56 | 1.10 (1.07–1.14) |
| 9 | *HNF4A* | ENSG00000101076 | rs6017317 | 20 | G/T | 0.48 | 1.09 (1.07–1.12) |
| 10 | *ANK1* | ENSG00000029534 | rs515071 | 8 | C/T | 0.81 | 1.18 (1.12–1.25) |
| 11 | *GRK5* | ENSG00000198873 | rs10886471 | 10 | C/T | 0.79 | 1.12 (1.08–1.16) |
| 12 | *RASGRP1* | ENSG00000172575 | rs7403531 | 15 | T/C | 0.33 | 1.10 (1.06–1.13) |
| 13 | *PAX4* | ENSG00000106331 | rs10229583 | 7 | G/A | 0.83 | 1.14 (1.09, 1.19) |
| 14 | *GPSM1* | ENSG00000160360 | rs11787792 | 9 | A/G | 0.87 | 1.15 (1.10, 1.20) |
| 15 | *SLC16A13* | ENSG00000174327 | rs312457 | 17 | G/A | 0.08 | 1.20 (1.14, 1.26) |
| 16 | *ST6GAL1* | ENSG00000073849 | rs16861329 | 3 | G/A | 0.75 | 1.09 (1.06–1.12) |
| 17 | *VPS26A* | ENSG00000122958 | rs1802295 | 10 | A/G | 0.26 | 1.08 (1.05–1.12) |
| 18 | *HMG20A* | ENSG00000140382 | rs7178572 | 15 | G/A | 0.52 | 1.09 (1.06–1.12) |
| 19 | *AP3S2* | ENSG00000157823 | rs2028299 | 15 | C/A | 0.31 | 1.10 (1.07–1.13) |
| 20 | *HNF4A* | ENSG00000101076 | rs4812829 | 20 | A/G | 0.29 | 1.09 (1.06–1.12) |
| 21 | *SGCG* | ENSG00000102683 | rs9552911 | 13 | G/A | 0.92 | 1.49 (1.30–1.72) |
| 22 | *TMEM163* | ENSG00000152128 | rs6723108 | 2 | T/G | 0.86 | 1.31 (1.20–1.44) |
| 23 | *GRB14* | ENSG00000115290 | rs3923113 | 2 | C/A | 0.74 | 1.09 (1.07–1.29) |

**Table S2. Transcription factor enrichment**

| **TF Name** | **TF-SNP matches (genome-wide)** | | **Fraction of TF-SNP hits** | **Enrichment** | **P-value** |
| --- | --- | --- | --- | --- | --- |
| Rfx1 | 7546 | 0.000132521 | | 113.7291943 | 0.008763867 |
| Nkx2-5 | 8744 | 0.000114364 | | 98.14735819 | 0.010149898 |
| NR2C2 | 19125 | 5.23E-05 | | 44.87322876 | 0.02209946 |
| MZF1_5-13 | 23898 | 4.18E-05 | | 35.91097581 | 0.027557199 |
| MZF1_1-4 | 126853 | 7.88E-06 | | 6.765314971 | 0.139819526 |
| ZNF263 | 264957 | 3.77E-06 | | 3.239018029 | 0.274795188 |
| ARID3A | 187194 | 0 | | 0 | 1 |
| Arnt | 50634 | 0 | | 0 | 1 |
| Arnt_Ahr | 75959 | 0 | | 0 | 1 |
| Atoh1 | 16034 | 0 | | 0 | 1 |
| BATF_JUN | 5193 | 0 | | 0 | 1 |
| BRCA1 | 29963 | 0 | | 0 | 1 |
| Bach1_Mafk | 11016 | 0 | | 0 | 1 |
| Bcl6 | 10619 | 0 | | 0 | 1 |
| Bhlhe40 | 10582 | 0 | | 0 | 1 |
| CDX2 | 12537 | 0 | | 0 | 1 |
| CEBPA | 4936 | 0 | | 0 | 1 |
| CEBPB | 12839 | 0 | | 0 | 1 |
| CREB1 | 2289 | 0 | | 0 | 1 |
| CTCF | 19639 | 0 | | 0 | 1 |
| Crx | 4969 | 0 | | 0 | 1 |
| DUX4 | 5218 | 0 | | 0 | 1 |
| Ddit3_Cebpa | 5085 | 0 | | 0 | 1 |
| E2F1 | 16499 | 0 | | 0 | 1 |
| E2F3 | 39019 | 0 | | 0 | 1 |
